# Supplementary material for: Heat adaptation of phage T7 under an extended genetic code
Source: Virus Evol. 2021 Dec 1;7(2):veab100. doi: 10.1093/ve/veab100 (PMC8923235; doi:10.1093/ve/veab100)
Supplement: veab100_Supp [file veab100_supp.zip › Sequencing_Analysis.zip!/Sequencing_Analysis/S2/evidence/MC_SIDE_1_38.html]

BRESEQ :: Evidence


| Predicted mutation | | | | | | |
| --- | --- | --- | --- | --- | --- | --- |
| evidence | seq id | position | mutation | annotation | gene | description |
| MC JC | NC\_001604.1 | 1,253 | Δ1,489 bp | intergenic (–/–) | – / – | –/– |

| Missing coverage evidence... | | | | | | | | | | |
| --- | --- | --- | --- | --- | --- | --- | --- | --- | --- | --- |
|  |  |  | seq id | start | end | size | ←reads | reads→ | gene | description |
| \* | \* | ÷ | NC\_001604.1 | 1253 | 2741 | 1489 | 5746 [2] | [2] 5753 | *–/–* | –/– |

| New junction evidence | | | | | | | | | | | |
| --- | --- | --- | --- | --- | --- | --- | --- | --- | --- | --- | --- |
|  | | seq id | position | reads (cov) | reads (cov) | score | skew | freq | annotation | gene | product |
| \* | ? | NC\_001604.1 | = 1252 | 2 (0.000) | 5642 (1.100) | 443/580 | 0.0 | 100.0% | intergenic (–/–) | *–/–* | –/– |
|  | ? | NC\_001604.1 | 2742 = | 0 (0.000) |  |  |  |  | intergenic (–/–) | *–/–* | –/– |

Only 100 of 5746 total aligned reads displayed.  

`AACGTTTTCGACCACGCTTACGAAATGCTGAAAGAAAACATCCGTTATGATGACATCCGTGACACTGATGACCTGCACGATGCTATTCACATGGCTGCCGATAATGCAGTTCCGCACTACTACGCTGACATCTTTAGCGTAATGGCAAGTGAGGGCATTGACCTTGAGTTCGAAGACTCTGGTCTGATGCCTGACACCAAGGACGTAATCCGCATCCTGCAAGCGCGTATCTATGAGCAATTAACGATTGACCTCTGGGAAGACGCAGAAGACTTGCTCAATGAATACTTGGAGGAAGTCGAGGAGTACGAGGAGGATGAAGAGTAATGTCTACTACCAACGTGCAATACGGTCTGACCGCTCAAACTGTACTTTTCTATAGCGACATGGTGCGCTGTGGCTTTAACTGGTCACTCGCAATGGCACAGCTCAAAGAACTGTACGAAAACAACAAGGCAATAGCTTTAGAATCTGCTGAGTGATAGACTCAAGGTCGCTCCTAGCGAGTGGCCTTTATGATTATCACTTTACTTATGAGGGAGTAATGTATATGCTTACTATCGGTCTACTCACCGCTCTAGGTCTAGCTGTAGGTGCAT  >  NC_001604.1/952‑1550`  

                                                                                                                                                                                                                                                                                                            `|`                                                                                                                                                                                                                                                                                                            
                `cTTACGAAATGCTGAAAGAAAACATCCGTTATGATGACATCCGTGACACTGATGACCTGCACGATGCTATTCACATGGCTGCCGATAATGCAGTTCCGCACTACTACGCTGACATCTTTAGCGTAATGGCAAGTGAGGGCATTGACCTTGAGTTCGAAGACTCTGGTCTGATGCCTGACACCAAGGACGTAATCCGCATCCTGCAAGCGCGTATCTATGAGCAATTAACGATTGACCTCTGGGAAGACGCAGAAGACTTGCTCAATGAATACTTGGAGGAAGTCGcacgacagaaagaaat                                                                                                                                                                                                                                                                                            <  1:257640‑M1/301‑17 (MQ=255)`  
                `cTTACGAAATGCTGAAAGAAAACATCCGTTATGATGACATCCGTGACACTGATGACCTGCACGATGCTATTCACATGGCTGCCGATAATGCAGTTCCGCACTACTACGCTGACATCTTTAGCGTAATGGCAAGTGAGGGCATTGACCTTGAGTTCGAAGACTCTGGTCTGATGCCTGACACCAAGGACGTAATCCGCATCCTGCAAGCGCGTATCTATGAGCAATTAACGATTGACCTCTGGGAAGACGCAGAAGACTTGCTCAATGAATACTTGGAGGAAGTCGcacgacagaaagaaat                                                                                                                                                                                                                                                                                            <  1:707335‑M1/301‑17 (MQ=255)`  
                                     `aCATCCGTTATGATGACATCCGTGACACTGATGACCTGCACGATGCTATTCACATGGCTGCCGATAATGCAGTTCCGCACTACTACGCTGACAACTTTAGCGTAATGGCAAGTGAGGGCATTGACCTTGAGTTCGAAGACTCTGGTCTGATGCCTGACACCAAGGACGTAATCCGCATCCTGCAAGCGCGTATCTATGAGCAATTAACGATTGACCTCTGGGAAGACGCAGAAGACTTGCTCAATGAATACTTGGAGGAAGTCGcacgacagaaagaaattgaccgcgctaaggcccgtaa                                                                                                                                                                                                                                                                       <  1:79136‑M1/301‑38 (MQ=255)`  
                                      `cATCCGTTATGATGACATCCGTGACACTGATGACCTGCACGATGCTATTCACATGGCTGCCGATAATGCAGTTCCGCACTACTACGCTGACATCTTTAGCGTAATGGCAAGTGAGGGCATTGACCTTGAGTTCGAAGACTCTGGTCTGATGCCTGACACCAAGGACGTAATCCGCATCCTGCAAGCGCGTATCTATGAGCAATTAACGATTGACCTCTGGGAAGACGCAGAAGACTTGCTCAATGAATACTTGGAGGAAGTCGcacgacagaaagaaattgaccgcgctaaggcccgtaaa                                                                                                                                                                                                                                                                      >  1:806335‑M1/1‑263 (MQ=255)`  
                                       `aTCCGTTATGATGACATCCGTGACACTGATGACCTGCACGATGCTATTCACATGGCTGCCGATAATGCAGTTCCGCACTACTACGCTGACATCTTTAGCGTAATGGCAAGTGAGGGCATTGACCTTGAGTTCGAAGACTCTAGTCTGATGCCTGACACCAAGGACGTAATCCGCATCCTGCAAGCGCGTATCTATGAGCAATTAACGATTGACCTCTGGGAAGACGCAGAAGACTTGCTCAATGAATACTTGGAGGAAGTCGcacgacagaaagaaattgaccgcgctaaggcccgtaaag                                                                                                                                                                                                                                                                     <  1:112825‑M1/301‑40 (MQ=255)`  
                                        `acagttaTGATGACATCCGTGACACTGATGACCTGCACGATGCTATTCACATGGCTGCCGATAATGCAGTTCCGAACTACTACGCTGACATCTTTAGCGTAATGGCAAGTGAGGGCATTGACCTTGAGTTCGAAGACTCTGGTCTGATGCCTGACACCAAGGACGTAATCCGCATCCTGCAAGCGCGTATCTATGAGCAATTAACGATTGACCTCTGGGAAGACGCAGAAGACTTGCTCAATGAATACTTGGAGGAAGTCGcacgacagaaagaaattgaccgcgctaaggcccgtaaaga                                                                                                                                                                                                                                                                    <  1:587410‑M1/298‑41 (MQ=255)`  
                                                        `ccGTGACACTGATGACCTGCACGATGCTATTCACATTGCTGCCGATAATGCAGTTCCGCACTACTACGCTGACATCTTTAGCGTAATGGCAAGTGAGGGCATTGACCTTGAGTTCGAAGACTATGGTCTGATGCCTGACACCAAGGACGTAATCCGCATCCTGCAAGCGCGTATCTATGAGCAATTAACGATTGACCTCTGGGAAGACGAAGAAGACTTGCTCAATGAATACTTGGAGGAAGTCGcacgacagaaagaaattgacagcgataaggcccgtcaagaacgtcacgaggggcgc                                                                                                                                                                                                                                                    >  1:5952‑M1/1‑245 (MQ=255)`  
                                                             `acacTGATGACCTGCACGATGCTATTCACATGGCTGCCGATAATGCAGTTCCGCACTACTACGCTGACATCTGTAGCGTAATGGCAAGTGAGGGCATTGACCTTGAGTTCGAAGACTCTGGTCTGATGCCTGACACCAAGGACGTAATCCGCATCCTGCAAGCGCGTATCTATGAGCAATTAACGATTGACCTCTGGGAAGACGCAGAAGACTTGCTCAATGAATACTTGGAGGAAGTCGcacgacagaaagaaattgaccgcgctacggcccgtaaagaacgtcacgaggggcgcttaga                                                                                                                                                                                                                                               >  1:371623‑M1/1‑240 (MQ=255)`  
                                                             `acacTGATGACCTGCACGATGCTATTCACATGGCTGCCGATAATGCAGTTCCGCACTACTACGCTGACATCTATAGCGCAATGACAAGTGAGGACATTGACCTTGAGTACGAAAACTCTTGTCTGATGTATGACACCAAGGACGTAATCCGCATCCTGCAAGCGCGTATCTATGAGCAATTAACGAGTGACCTCAGTGAAGACGCAGAAGACTTGCACAAAGAATACGTGGAGGAAGTCGcacgacagaaagtaattgctcgcgctaagccccgtaaatgtctcatatacaaatatccgag                                                                                                                                                                                                                                               >  1:765133‑M1/1‑240 (MQ=255)`  
                                                                `cTGATGACCTGCACGATGCTATTCACATGGCTGCCGATAATGCAGTTCCGCACTACTACGCTGACATCTTTAGCGTAATGGCAAGTGAGGGCATTGACCTTGAGTTCGAAGACTCTGGTCTGATGCCTGACACCAAGGACGTAATCCGCATCCTGCAAGCGCGTATCTATGAGCAATTAACGATTGACCTCTGGGAAGACGCATAAGACTTGCTCAATGAATACTTGGAGGAAGTCGcacgacagaaagaaattgaccgcgctaaggcccgtaaagaacgtcacgaggggcgcttagaggc                                                                                                                                                                                                                                            >  1:673171‑M1/1‑237 (MQ=255)`  
                                                                  `gatgaCCTGCACGATGCTATTCACATGGCTGCCGATAATGCAGTTCCGCACTACTACGCTGACATCTTTAGCGTAATGGCAAGTGAGGGCATTGACCTTGAGTTCGAAGACTCTGGTCTGATGCCTGACACCAAGGACGTAATCCGCATCCTGCAAGCGCGTATCTATGAGCAATTAACGATTGACCTCTGGGAAGACGCAGAAGACTTGCTCAATGAATACTTGGAGGAAGTCGcacgacagaaagaaattgaccgcgctaaggcccgtaaagaacgtcacgaggggcgcttagaggcac                                                                                                                                                                                                                                          >  1:779166‑M1/1‑235 (MQ=255)`  
                                                                  `gatgaCCTGCACGATGCTATTCACATGGCTGCCGATAATGCAGTTCCGCACTACTACGCTGACATCTTTAGCGTAATGGCAAGTGAGGGCATTGACCTTGAGTTCGAAGACTCTGGTCTGATGCCTGACACCAAGGACGTAATCCGCATCCTGCAAGCGCGTATCTATGAGCAATTAACGATTGACCTCTGGGAAGACGCAGAAGACTTGCTCAATGAATACTTGGAGGAAGTCGcacgacagaaagaaattgaccgcgctaaggcccgtaaagaacgtaacgaggggcgcttagaggcag                                                                                                                                                                                                                                          >  1:479061‑M1/1‑235 (MQ=255)`  
                                                                          `gCACGATGCTATTCACATGGCTGCCGATAATGCAGTTCCGCACTACTACGCTGACATCTTTAGCGTAATGGCAAGTGAGGGCATTGACCTTGAGTTCGAAGACTCTGGTCTGATGCCTGACACCAAGGACGTAATCCGCATCCTGCAAGCGCGTATCTATAAGCAATTAACGATTGACCTCTGGGAAGACGCAGAAGACTTGCTCAATGAATACTTGGAGGAAGTCGcacgacagaaagaaattgaccgcgctaaggcccgtaaagaacgtcacgaggggcgcttagaggcacgcagattc                                                                                                                                                                                                                                  >  1:317653‑M1/1‑227 (MQ=255)`  
                                                                               `aTGCTATTCACATTGCTGCCGATAATGCAGTTCCGCACTACTACGCTGACATCTTTAGCGTAATGGCAAGTGAGGGCATTGACCTTGAGTTCGAAGACTCTGGTCTGATGCCTGACACCAAGGACGTAATCCGCATCCTGCAAGCGCGTATCTATGAGCAATTAACGATTGACCTCTGGGAAGACGCAGAAGACTTGCTCAATGAATACTTGGCGGAAGTCGcacgacagaaagacattgaccgcgctaaggcccgtaaagaacgtcacgcggggcgcttagcggcccgcagattcaaccg                                                                                                                                                                                                                             >  1:178086‑M1/1‑222 (MQ=255)`  
                                                                               `aTGCTATTCACATGGCTGCCGATAATGCAGTTCCGCACTACTACGCTGACATCTTTAGCGTAATGGCAAGTGAGGGCATTGACCTTGAGTTCGAAGACTCTGGTCTGATGCCTGACACCAAGGACGTAATCCGCATCCTGCAAGCGCGTATCTATGAGCAATTAACGATTGACCTCTGGGAAGACGCAGAAGACTTGCTCAATGAATACTTGGAGGAAGTCGcacgacagaaagaaattgaccgcgctaaggcccgtaaagaacgtcacgaggggcgcttagaggcacgcagattcaaacg                                                                                                                                                                                                                             >  1:431051‑M1/1‑222 (MQ=255)`  
                                                                               `aTGCTATTCACATGGCTGCCGATAATGCAGTTCCGCACTACTACGCTGACATCTTTAGCGTAATGGCAAGTGAGGGCATTGACCTTGAGTTCGAAGACTCTGGTCTGATGCCTGACACCAAGGACGTAATCCGCATCCTGCAAGCGCGTATCTATGAGCAATTAACGATTGACCTCTGGGAAGACGCAGAAGACTTGCTCAATGAATACTTGGAGGAAGTCGcacgacagaaagaaattgaccgcgctaaggcccgtaaagaacgtcacgaggggcgcttagaggcacgcagattcaaacg                                                                                                                                                                                                                             >  1:294938‑M1/1‑222 (MQ=255)`  
                                                                                    `aTTCACATGGCTGCCGATAATGCAGTTCCGCACTACTACGCTGACATCTTTAGCGTAATGGCAAGTGAGGGCATTGACCTTGAGTTCGAAGACTCTTGTCTGATGCCTGACACCAAGGACGTAATCCGCATCCTGCAAGCGCGTATCTATGAGCAATTAACGATTGACCTCTGAGAAGACGCAGAAGACTTGCTCAATGAATACTTGTAGGAAGTTGcacgacagaaagaaattgaccgcgctaaggcccgtaaagaacgtcacgagggtcgattagaggaccgccgattcaaacggcgca                                                                                                                                                                                                                        >  1:455181‑M1/1‑217 (MQ=255)`  
                                                                                    `aTTCACATGGCTGCCGATAATGCAGTTCCGCACTACTACGCTGACATCTTTAGCGTAATGGCAAGTGAGGGCATTGACCTTGAGTTCGAAGACTCTGGTCTGATGCCTGACACCAAGGACGTAATCCGCATCCTGCAAGTGCGTATCTATGAGCAATTAACGATTGACCTCTGGGAAGACGCAGAAGACTTGCTCAATGAATACTTGGAGGAAGTCGcacgacagaaagaaattgaccgcgctaaggcccgtaaagaacgtcacgaggggcgcttagaggcacgcagattcaaacgtcgca                                                                                                                                                                                                                        >  1:757503‑M1/1‑217 (MQ=255)`  
                                                                                    `aTTCACATGGCTGCCGATAATGCAGTTCCGCACTACTACGCTGACATCTTTAGCGTAATGGCAAGTGAGGGCATTGACCTTGAGTTCGAAGACTCTGGTCTGATGCCTGACACCAAGGACGTAATCCGCATCCTGCAAGCGCGTATCTATGAGCAATTAACGATTGACCTCTGGGAAGACGCAGAAGACTTGCTCAATGAATACTTGGAGGAAGTCGcacgacagaaagaaattgaccgcgctaaggcccgtaaagaacgtcacgaggggcgcttagaggcacgcagattcaaacgtcgca                                                                                                                                                                                                                        >  1:83331‑M1/1‑217 (MQ=255)`  
                                                                                                      `tgtataagagacagctactacgctgacatctttaGCGTAATGGCAAGTGAGGGCATTGACCTTGAGTTCGAAGACTCTGGTCTGATGCCTGACACCAAGGACGTAATCCGCATCCTGCAAGCGCGTATCTATGAGCAATTAACGATTGACCTCTGGGAAGACGCAGAAGACTTGCTCAATGAATACTTGGAGGAAGTCGcacgacagaaagaaattgaccgcgctaaggcccgtaaagaacgtcacgaggggcgcttagaggcacgcagattcaaacgtcgcaaccgcaaggcacgtaaag                                                                                                                                                                                                      <  1:447161‑M1/287‑103 (MQ=255)`  
                                                                                                         `gCAGTTCCGCACTACTACGCTGACATCTTTAGCGTAATGGCAAGTGAGGGCATTGACCTTGAGTTCGAAGACTCTGGTCTGATGCCTGACACCAAGGACGTAATCCGCATCCTGCAAGCGCGTATCTATGAGCAATTAACGATTGACCTCTGGGAAGACGCAGAAGACTTGCTCAATGAATACTTGGAGGAAGTCGcacgacagaaagaaattgaccgcgctaaggcccgtaaagaacgtcacgaggggcgcttagaggcacgcagattcaaacgtcgcaaccgcaaggcacgtaaagcac                                                                                                                                                                                                   <  1:18693‑M1/301‑106 (MQ=255)`  
                                                                                                            `gTTCCGCACTACTACGCTGACATCTTTAGCGTAATGGCAAGTGAGGGCATTGACCTTGAGTTCGAAGACTCTGGTCTGGTGCCTGACACCAAGGACGTAATCCGCATCCTGCAAGCGCGTATCTATGCGCAATTAACGATTGACCTCTGGGAAGACGCAGAAGACTTGCTCAATGAATACTTGGAGGAAGTCGcacgacagcaagaaattgaccgcgctaaggcccgtaaagaacgtcacgaggggcgcttagaggcacgcagattccaacgtcccacacgcaaggcacgtaaagaacaca                                                                                                                                                                                                >  1:157604‑M1/1‑193 (MQ=255)`  
                                                                                                               `ccGCACTACTACGCTGACATCTTTAGCGTAATGGCAAGTGAGGGCATTGACCTTGAGTTCGAAGACTCTGGTCTGATGCCTGACACCAAGGACGTAATCCGCATCCTGCAAGCGCGTATCTATGAGCAATTAACGATTGACCTCTGGGAAGACGCAGAAGACTTGCTCAATGAATACTTGGAGGAAGTCGcacgacagaaagcaattgaccgcgctaaggcccgtaaagcacgtcacgaggggcgcttagaggcacgcatattcaaacgtcgccaccgcaaggcacctaaagcacactaag                                                                                                                                                                                             >  1:713814‑M1/1‑190 (MQ=255)`  
                                                                                                                    `ctactaCGCTGACATCTTTAGCGTAATGGCAAGTGAGGGCATTGACCTTGAGTTCGAAGACTCTGGTCTGATGCCTGACACCAAGGACGTAATCCGCATCCTGCAAGCGCGTATCTATGAGCAATTAACGATTGACCTCTGGGAAGACGCAGAAGACTTGCTCAATGAATACTTGGAGGAAGTCGcacgacagaaagaaattgaccgcgctaaggcccgtaaagaacgtcacgaggggcgcttagaggcacgcagattcaaacgtggcaaccgcaaggcacgtaaagcacaacaagctaag                                                                                                                                                                                        >  1:522725‑M1/1‑185 (MQ=255)`  
                                                                                                                    `ctactaCGCTGACATCTTTAGCGTAATGGCAAGTGAGGGCATTGACCTTGAGTTCGAAGACTCTGGTCTGATGCCTGACACCAAGGACGTAATCCGCATCCTGCAAGCGCGTATCTATGAGCAATTAACGATTGACCTCTGGGAAGACGCAGAAGACTTGCTCAATGAATACTTGGAGGAAGTCGcacgacagaaagaaattgaccgcgctaaggcccgtaaagaacgtcacgaggggcgcttagaggcacgcagattcaaacgtcgcaaccgcaaggcctgtctcttatacacatctccc                                                                                                                                                                                        >  1:799936‑M1/1‑185 (MQ=255)`  
                                                                                                                    `ctactaCGCTGACATCTTTAGCGTAATGGCAAGTGAGGGCATTGACCTTGAGTTCGAAGACTCTGGTCTGATGCCTGACACCAAGGACGTAATCCGCATCCTGCAAGCGCGTATCTATGAGCAATTAACGATTGACCTCTGGGAAGACGCAGAAGACTTGCTCAATGAATACTTGGAGGAAGTCGcacgacagaaagaaattgaccgcgctaaggcccgtaaagaacgtcacgaggggcgcttagaggcacgcagattcaaacgtcgcaaccgcaaggcacgtaaagcacacaaagctaag                                                                                                                                                                                        >  1:213122‑M1/1‑185 (MQ=255)`  
                                                                                                                    `ctactaCGCTGACATCTTTAGCGTAATGGCAAGTGAGGGCATTGACCTTGAGTTCGAAGACTCTGGTCTGATGCCTGACACCAAGGACGTAATCCGCATCCTGCAAGAGCGTATCTATGAGCAATTAACGATTGACCTCTGGGAAGACGCAGAAGACTTGCTCAATGAATACTTGGAGGAAGTCGcacgacagaaagaaattgaccgcgctaaggcccgtaaagaacgtcacgaggggcgcttagaggcacgcagattcaaacgtcgcaaccgcaaggcacgtaaagcacacaaagcctgt                                                                                                                                                                                        >  1:324816‑M1/1‑185 (MQ=255)`  
                                                                                                                      `actacGCTGACATCTTTAGCGTAATGGCAAGTGAGGGCATTGACCTTGAGTTCGAAGACTCTGGTCTGATGCCTGACACCAAGGACGTAATCCGCATCCTGCAAGCGCGTATCTATGAGCAATTAACGATTGACCTCTGGGAAGACGCAGAAGACTTGCTCAATGAATACTTGGAGGAAGTCGcacgacagaaagaaattgaccgcgctaaggcccgtaaagaacgtcacgaggggcgcttagaggcacgcagattcaaacgtcgcaaccgcaaggcacgtaaagcacacaaagcccgtcc                                                                                                                                                                                      >  1:350017‑M1/1‑183 (MQ=255)`  
                                                                                                                      `actacGCTGACATCTTTAGCGTAATGGCAAGTGAGGGCATTGACCTTGAGTTCGAAGACTCTGGTCTGATGCCTGACACCAAGGACGTAA‑CCGCATCCTGCAAGCGCGTATCTATGAGCAATTAACGATTGACCTCTGGGAAGACGCAGAAGACTTGCTCAATGAATACTTGGATGAAGTCGcactacagaaagaaattgaccgcgctaaggcccgtaaagaacgtcacgaggggcgcttagaggcacgcagattcaaacgtcgcaaccgcctgtctcttatacacatctccgagcccacg                                                                                                                                                                                     >  1:625539‑M1/1‑182 (MQ=255)`  
                                                                                                                         `acGCTGACATCTTTAGCGTAATGGCAAGTGAGGGCATTGACCTTGAGTTCGAAGACTCTGGTCTGATGCCTGACACCAAGGACGTAATCCGCATCCTGCAAGCGCGTATCTATGAGCAATTAACGATTGACCTCTGGGAAGACGCAGAAGACTTGCTCAATGAATACTTGGAGGAAGTCGcacgacagaaagaaattgaccgcgctaaggcccgtaaagaacgtcacgaggggcgcttagaggcacgcagattcaaacgtcgcaaccgcaaggcacgtaaagcacacaaagctaagcgcga                                                                                                                                                                                   >  1:573189‑M1/1‑180 (MQ=255)`  
                                                                                                                              `gACATCTTTAGCGTAATGGCAAGTGAGGGCATTGACCTTGAGTTCGAAGACTCTGGTCTGATGCCTGACACCAAGGACGTAATCCGCATCCTGCAAGCGCGTATCTATGAGCAATTAACGATTGACCTCTGGGAAGACGCAGAAGACTTGCTCAATGAATACTTGGAGGAAGTCGcacgacagaaagaaattgaccgcgctaaggcccgtaaagaacgtcacgaggggcgcttagaggcacgcagattcaaacgtcgcaaccgcaaggcacgtaaagcacacaaagctaagcgcgaacgaa                                                                                                                                                                              >  1:106593‑M1/1‑175 (MQ=255)`  
                                                                                                                              `gACATCTTTAGCGTAATGGCAAGTGAGGGCATTGACCTTGAGTTCGAAGACTCTGGTCTGATGCCTGACACCAAGGACGTAATCCGCATCCTGCAAGCGCGTATCTATGAGCAATTAACGATTGACATCTGGGAAGACGCAGAAGAGTTGCTCAATGAATACTTGGAGGAAGTCGcacgacagaaagaaattgaccgcgctatggcccgtgaagaacgtcacgaggggggattagagggacgcagattcaaacgtcgcaacaggaaggcacgtaaagcacacaaagctaagggcgacagaa                                                                                                                                                                              >  1:681729‑M1/1‑175 (MQ=255)`  
                                                                                                                                 `aTCTTTAGCGTAATGGCAAGTGAGGGCATTGACCTTGAGTTCGAAGACTCTGGTCTGATGCCTGACACCAAGGACGTAATCCGCATCCTGCAAGCGCGTATCTATGAGCAATTAACGATTGACCTCTGGGAAGACGCAGAAGACTTGCTCAATGAATACTTGGAGGAAGTCGcacgacagaaagaaattgaccgcgctaaggcccgtaaagaacgtcacgaggggcgcttagaggcacgcagattcaaacgtcgcaaccgcaaggcacgtaaagcacacaaagctaagcgcgaaagaatgc                                                                                                                                                                           <  1:378866‑M1/301‑130 (MQ=255)`  
                                                                                                                                   `cTTTAGCGTAATGGCAAGTGAGGGCATTGACCTTGAGTTCGAAGACTCTGGTCTGATGCCTGACACCAAGGACGTAATCCGCATCCTGCAAGCGCGTATCTATGAGCAATTAACGATTGACCTCTGGGAAGACGCAGCAGACTTGCTCAATGAATACTTGGAGGAAGTCGcacgacagaaagaaattgaccgcgctaaggcccgtaaagaacgtcacgaggggcgcttagaggcacgcagattcaaacgtcgcaaccgcaaggcacgtaaagcacacaaagctaagcgcgaaagaatgctt                                                                                                                                                                         >  1:333202‑M1/1‑170 (MQ=255)`  
                                                                                                                                        `gCGTAATGGCAAGTGAGGGCATTGACCTTGAGTTCGAAGACTCTGGTCTGATGCCTGACACCAAGGACGTAATCCGCATCCTGCAAGCGCGTATCTATGAGCAATTAACGATTGACCTCTGGGAAGACGCAGAAGACTTGCTCAATGAATACTTGGAGGAAGTCGcacgacagaaagaaatttaccgcgctaaggcccgtaaagaacgtcacgaggggcgcttataggcacgcagattcaaacgtcgaaaccgcaaggcacggaaagcacacaaagataagcgcgaaagaatgcttgctgc                                                                                                                                                                    >  1:771898‑M1/1‑165 (MQ=255)`  
                                                                                                                                             `aTGGCAAGTGAGGGCATTGACCTTGAGTTCGAAGACTCTGGTCTGATGCCTGACACCAAGGACGTAATCCGCATCCTGCAAGCGCGTATCTATGAGCAATTAACGATTGATCTCTGGGAAGACGCAGAAGACTTGCTCAATGAATACTTGGAGGAAGTCGcacgacagaaagaaattgaccgcgctaaggcccgtaaagaacgtcacgaggggcgcttagaggcacgcagattcaaacgtcgcaaccgcaaggcacgtaaagcacacaaagctaagcgcgaaagaatgcttgctgcgtggc                                                                                                                                                               >  1:666036‑M1/1‑160 (MQ=255)`  
                                                                                                                                             `aTGGCAAGTGAGGGCATTGACCTTGAGTTCGAAGACTCTGGTCTGATGCCTGACACCAAGGACGTAATCCGCATCCTGCAAGCGCGTATCTATGAGCAATTAACGATTGACCTCTGGGAAGACGCAGAAGACTTGCTCAATGAATACTTGGAGGAAGTCGcacgacagaaagaaattgaccgcgctaaggcccgtaaagaacgtcacgaggggcgcttagaggcacgcagattcaaacgtcgcaaccgcaaggcacgtaaagcacacaaagctaagcgcgaaagaatgcttgctgcgtggg                                                                                                                                                               >  1:820743‑M1/1‑160 (MQ=255)`  
                                                                                                                                               `gtataagagacagattgaccttgagttCGAAGACTCTGGTCTGATGCCTGACACCAAGGACGTAATCCGCATCCTGCAAGCGCGTATCTATGAGCAATTAACGATTGACCTCTGGGAAGACGCAGAAGACTTGCTCAATGAATACTTGGAGGAAGTCGcacgacagaaagaaattgaccgcgctaaggcccgtaaagaacgtcacgaggggcgcttagaggcacgcagattcaaacgtcgcaaccgcaaggcacgtaaagcacacaaagctaagcgcgaaagaatgcttgctgcgtggcga                                                                                                                                                             <  1:100099‑M1/288‑144 (MQ=255)`  
                                                                                                                                               `ggCAAGTGAGGGCATTGACCTTGAGTTCGAAGACTCTGGTCTGATGCCTGACACCAAGGACGTAATCCGCATCCTGCAAGCGCGTATCTATGAGCAATTAACGATTGACCTATGGGAAGACGCAGAAGACTTGCTCAATGAATACTTGGAGGAAGTCGcacgacagaaagaaattgaccgcgctaaggcccgtaaagaacgtcacgaggggcgcttagaggcacgcagattcaaacgtcgcaaccgcaaggcacgtaaagcacacaaagctaagcgcgaaagaatgcttgctgcgtggcga                                                                                                                                                             >  1:280015‑M1/1‑158 (MQ=255)`  
                                                                                                                                                         `tgcATTGACCTTGAGTTCGAAGACTCTGGTCTGATGCCTGACACCAAGGACGTAATCCGCATCCTGCAAGCGCGTATCTATTAGCAATTAACGATTGACCTCTGGGAAGACGCAGAAGACTTGCTCAATGAATACTTGGAGGAAGTCGcacgacagaaagaaattgaccgcgctaaggcccgtaaagaacgtcacgaggggcgcttagaggcacgcagattcaaacgtcgcaaccgcaaggcacgtaaagcacacaaagctaagcgcgaaagaatgcttgctgcgtggcgatgggctgaac                                                                                                                                                   <  1:698534‑M1/300‑154 (MQ=255)`  
                                                                                                                                                         `ggCATTGACCTTGAGTTCGAAGACTCTGGTCTGATGCCTGACACCAAGGACGTAATCCGCATCCTGCAAGCGCGTATCTATGAGCAATTAACGATTGACCTCTGGGAAGACGCAGAAGACTTGCTCAATGAATACTTGGAGGAAGTCGcacgacagaaagaaattgaccgcgctaaggccagtaaagaacgtcacgaggggcgcttagaggcacgcagattcaaacgtcgcaaccgcaaggcacgtaaagaacacaaagctaagcgcgaaagaatgcttgctgcgtggcgatgggctgaac                                                                                                                                                   >  1:386498‑M1/1‑148 (MQ=255)`  
                                                                                                                                                          `gCATTTACCTTTAGTTCTAAGACTCTGGGATGATGCCTGACCCCAAGGACGTAATCCGCATCCTGCAAGCGCGTATCTATAAGCAATTAACGATTGACCTCTGGGAAGACGCAGAAGACTTGCTCAATGAATACTTGGAGGAGGTCGcacgacagaaagaaattgaccgcgctaaggcccgtaaagaacgtcacgagtggcgcttagaggcacgcagattcaaacgtcgcaaccgcaaggcacgtaaagcacacaaagctaagcgcgaaagaatgcttgctgcgtggcgatgggctaaacg                                                                                                                                                  <  1:207612‑M1/301‑155 (MQ=255)`  
                                                                                                                                                           `cATTGACCTTGAGTTCGAAGACTCTGGTCTGATGCCTGACACCAAGGACGTAATCCGCATCCTGCAAGCGCGTATCTATGAGCAATTAACGATTAACCTCTGGGAAGACGCAGAAGACTTGCTCAATGAATACTTGGAGGAAGTCGcacgacagaaagaaattgaccgcgctaaggcccgtaaagaacgtcacgaggggcgcttagaggcacgcagattcaaacgtcgcaaccgcaaggcacgtaaagcacacaaagctaagcgcgaaagaatgcttgctgcgtggcgatgggctgaacgt                                                                                                                                                 <  1:736781‑M1/301‑156 (MQ=255)`  
                                                                                                                                                                 `ccTTGAGTTCGAAGACTCTGGTCTGATGCCTGACACCAAGGACGTAATCCGCATCCTGCAAGCGCGTATCTATGAGCAATTAACGATTGACCTCTGGGAAGACGCAGAAGACTTGCTCAATGAATACTTGGAGGAAGTCGcacgacagaaagaaattgaccgcgctaaggcccgtaaagaacgtcacgagggtcgcttagaggcacgcagattcaaacgtcgcaaccgcaaggcacgtaaagcacacaaagctaagcgcgaaagaatgcttgctgcgtggcgatgggctaaacgtctgtct                                                                                                                                           >  1:250281‑M1/1‑140 (MQ=255)`  
                                                                                                                                                                     `gAGTTCGAAGACTCTGGTCTGATGCCTGACACCAAGGACGTAATCCGCATCCTGCAAGCGCGTATCTATGAGCAATTAACGATTGACCTCTGGGAAGACGCAGAAGACTTGCTCAATGAATACTTGGAGGAAGTCGcacgacagaaagaaattgaccgcgctaaggcccgtaaagaacgtcacgaggggcgcttagaggcacgcagattcaaacgtcgcaaccgcaaggcacgtaaagcacacaaagctaagcgcgaaagaatgcttgctgcgtggcgatgggctgaacgtcaagaacctg                                                                                                                                       >  1:91582‑M1/1‑136 (MQ=255)`  
                                                                                                                                                                       `gTTCGAAGACTCTGGTCTGATGCCTGACACCAAGGACGTAATCCGCATCCTGCAAGCGCGTATCTATGAGCAATGAACGATTGACCTCTGGGAAGACGCAGAAGACTTGCTCAATTAATACTTGGAGGAAGTCGcacgacagaaagaaattgaccgcgctaaggcccgtaaagaacgtcacgaggggcgcttagaggcacggagattcaaacgtcgcaaccgcaaggcacgtaaagcacacaaagctaagcgcgaaagaatgcttgctgcgtggcgatgggctgaacgtcaagcacggcgg                                                                                                                                     >  1:243074‑M1/1‑134 (MQ=255)`  
                                                                                                                                                                           `gAAGACTCTGGTCTGATGCCTGACACCAAGGACGTAATCCGCATCCTGCAAGCGCGTATCTATGAGCAATTAACGATTGACCTCTGGGAAGACGCAGAAGACTTGCTCAATGAATACTTGGAGGAAGTCGcacgacagaaagaaattgaccgcgctaagtcccgtaaagaacgtcacgagggtcgcttagaggcacgcagattcaaacgtcgcaaccgcaaggcacgtaaagcacacaaagctaagcgcgaaagaatgcttgctgcgtggcgatgggctgaacgtcaagaacggcgtaacc                                                                                                                                 <  1:366043‑M1/301‑172 (MQ=255)`  
                                                                                                                                                                            `agagacagggtctgatgcCTGAACCCAAGGACGTAATCCGCATCCGGCAAGCGCGTATCTATGAGCAATTAACGATTGACCTCTGGGAAGACGCAGAAGACTTGCTCAATGAATACTTGGAGGAAGTCGcacgacagaaagaaattgaccgcgctaaggcccgtaaagaacgtcacgaggggcgcttagaggcacgcagattcaaacgtcgcaaccgcaaggcacgtaaagcacacaaagctaagcgcgaaagaatgcttgctgcgtggcgatgggctgaacgtcaagaacggcgtaacca                                                                                                                                <  1:81426‑M1/293‑173 (MQ=255)`  
                                                                                                                                                                            `aaGACTCTGGTCTGATGCCTGACACCAAGGACGTAATCCGCATCCTGCAAGCGCGTATCTATGAGCAATTAACGATTGACCTCTGGGAAGACGCATAAGACTTGCTCAATGAATACTTTGAGGAAGTCGcacgacagaaagaaattgaccgcgctaaggcccgtaaagaacgtcacgaggggcgcttagaggcacgcagattcaaacgtcgcaaccgcaaggcacgtaaagcacacaaagctaagcgcgaaagaatgcttgctgcgtggcgatgggctgaacgtcaagaacggcgtaacca                                                                                                                                <  1:6416‑M1/301‑173 (MQ=255)`  
                                                                                                                                                                                 `aagggtctGATGCCTGACACCAAGGACGTAATCCGCATCCTGCAAGCGCGTATCTATGAGCAATTAACGATTGACCTCTGGGAAGACGCAGAAGACTTGCTCAATGAATACTTGGAGGAAGTCGcacgacagaaagaaattgaccgcgctaaggcccgtaaagaacgtcacgaggggcgcttagaggcacgcagattcaaacgtcgcaaccgcaaggcacgtaaagcacacaaagctaagcgagaaagaatgcttgctgcgtggcgatgggctgaacgtcaagaacggcgtaaccatgagg                                                                                                                           <  1:120728‑M1/298‑178 (MQ=255)`  
                                                                                                                                                                                  `ctGGTCTGATGCCTGACACCAAGGACGTAATCCGTATCCTGCAAGCGCGTATCTATGAGCAATTAACGATTGACCTTTGGGAAGACGCAGAAGACTTGCTCAATGAATACTTGGAGGAAGTCGcacgacagaaagaaattgaccgcgctaaggcccgtaaagaacgtcacgaggggcgcttagaggcacgcagattcaaacgtcgcaaccgcaaggcacgtaaagcacacaaagctaagcgcgaaagaatgcttgctgcgtggcgatgggctgaacgtcaagaacggcgtaaccatgaggt                                                                                                                          <  1:128541‑M1/301‑179 (MQ=255)`  
                                                                                                                                                                                  `acagtctGATGCCTGACACCAAGGACGTAATCCGCATCCTGCAAGCGCGTATCTATGAGCAATTAACGATTGACCTCTGGGAAGACGCAGAAGACTTGCTCAATGAATACTTGGAGGAAGTCGcacgacagaaagaaattgaccgcgctaaggcccgtaaagaacgtcacgaggggcgcttagaggcacgcagattcaaacgtcgcaaccgcaaggcacgtaaagcacacaaagctaagcgcgaaagaatgcttgctgcgtggcgatgggctgaacgtcaagaacggcgtaaccatgaggt                                                                                                                          <  1:538042‑M1/298‑179 (MQ=255)`  
                                                                                                                                                                                    `ggTCTGATGCCTGACACCAAGGACGTAATCCGCATCCTGCAAGCGCGTATCTATGAGCAATTAACGATTGACCTCTGGGAAGACGCAGAAGACTTGCTCAATGAATACTTGGAGGAAGTCGcacgacagaaagaaattgaccgcgctaaggcccgtaaagaacgtcacgaggggcgcttagaggcacgcagattcaaacgtcgcaaccgcaaggcacgtaaagcacacaaagctaagcgcgaaagaatgcttgctgcgtggcgatgggctgaacgtcaagaacggcgtaaccatgagatag                                                                                                                        >  1:170968‑M1/1‑121 (MQ=255)`  
                                                                                                                                                                                      `tCTGATGCCTGACACCAAGGACGTAATCCGCATCCTGCAAGCGCGTATCTATGAGCAATTAACGATTGACCTCTGGGAAGACGCAGAAGACTTGCTCAATGAATACTTGGAGGAAGTCGcacgacagaaagaaattgaccgcgctaaggcccgtaaagaacgtcacgaggggcgcttagaggcacgcagattcaaacgtcgcaaccgcaaggcacgtaaagcacacaaagctaagcgcgaaagaatgcttgcttcgtggcgatgggctgaacgtcaagaacggcgtaaccatgaggtagct                                                                                                                      >  1:500100‑M1/1‑119 (MQ=255)`  
                                                                                                                                                                                         `ataagagacagccaaggacgtaatCCGCATCCTGCAAGCGCGTATCTATGAGCAATTAACGATTGACCTCTGGGAAGACGCAGAAGACTTGCTCAATGAATACTTGGAGGAAGTCGcacgacagaaagaaattgaccgcgctaaggcccgtaaagaacgtcacgaggggcgcttagaggcacgcagattcaaacgtcgcaaccgcaaggcacgtaaagcacacaaagctaagcgcgaaagaatgcttgctgcgtggcgatgggctgaacgtcaagaacggcgtaaccatgaggtagctgta                                                                                                                   <  1:439537‑M1/290‑186 (MQ=255)`  
                                                                                                                                                                                            `gCCTGACACCAAGGACGTAATCCGCATCCTGCAAGCGCGTATCTATGAGCAATTAACGATTGACCTCTGGGAAGACGCAGAAGACTTGCTCAATGAATACTTGGAGGAAGTCGcacgacagaaagaaattgaccgcgctaaggcccgtaaagaacgtcacgaggggcgcttagaggcacgcagattcaaacgtcgcaaccgcaaggcacgtaaagcacacaaagctaagcgcgaaagaatgcttgctgcgtggcgatgggctgaacgtcaagaacggcgtaaccatgaggtagctgtagat                                                                                                                >  1:163438‑M1/1‑113 (MQ=255)`  
                                                                                                                                                                                            `gCCTGACACCAAGGACGTAATCCGCATCCTGCAAGCGCGTATCTATGAGCAATTAACGATTGACCTCTGGGAAGACGCAGAAGACTTGCTCAATGAATACTTGGAGGAAGTCGcacgacagaaagaaattgaccgcgctaaggcccgtaaagaacgtcacgaggggcgcttagaggcacgcagattcaaacgtcgcaaccgcaaggcacgtaaagcacacaaagctaagcgcgaaagaatgcttgctgcgtggcgatgggctgaacgtcaagaacggcgtaaccatgaggtagctgcagaa                                                                                                                >  1:22226‑M1/1‑113 (MQ=255)`  
                                                                                                                                                                                            `gCCTGACACCAAGGACGTAATCCGCATCCTGCAAGCGCGTATCTATGAGCAATTAACGATTGACCTCTGGGAAGACGCAGAAGACTTGCTCAATGAATACTTGGAGGAAGTCGcacgacagaaagaaattgaccgagctaaggcccgtaaagaacgtcacgaggggcgcttagaggcacgcagattcaaacgtcgcaaccgcaaggcacgtaaagcacacaaagctaagcgcgaaagaatgcttgctgcgtggcgatgggctgaacgtcaagaacggcgtaaccatgaggtagcctgtctc                                                                                                                >  1:689496‑M1/1‑113 (MQ=255)`  
                                                                                                                                                                                            `gCCTGACACCAAGGACGTAATCCGCATCCTGCAAGCGCGTATCTATAAGCAATTAACGATTGACCTCTGGGAAGACGCAGAAGACTTGCTCAATGAATACTTGGAGGAAGTCGcacgacagaaagaaattgaccgcgctaaggcccgtaaagaacgtcacgaggggcgcttagaggcacgcagattcaaacgtcgcaaccgcaaggcacgtaaagcacacaaagctaagcgcgaaagaatgcttgctgcgtggcgatgggctgaacgtcaagaacggcgtaaccatgaggtagctgtagat                                                                                                                <  1:53023‑M1/301‑189 (MQ=255)`  
                                                                                                                                                                                             `ccTGACACCAAGGACGTAATCCGCATCCTGCAAGCGCGTATCTATGAGCAATTAACGATTGACCTCTGGGAAGACGCAGAAGACTTGCTCAATGAATACTTGGAGGAAGTCGcacgacagaaagaaattgaccgcgctaaggcccgtaaagaacgtcacgaggggcgcttagaggcacgcagattcaaacgtcgcaaccgcaaggcacgtaaagcacacaaagctaagcgcgaaagaatgcttgctgcgtggcgatgggctgaacgtcaagaacggcgtaaccatgaggtagctgtagatg                                                                                                               <  1:142982‑M1/301‑190 (MQ=255)`  
                                                                                                                                                                                                `gACACCAAGGACGTAATCGGTCTCCTGCAAGCTCGTATCTATGAGCAATTAACGATTGACCTCTGGGAAGACGCAGAAGACTTGCTCAATGATTACTTGGAGGAAGTCGcacgacagaaagaaattgacggcgctaaggcccgtaaagaacgtaacgaggggcgcttagaggcacgcagattcaaacgtcgcaaccgcaaggcacgtaaagcacacaaagctaagcgcgaaagaattcttgctgcgtggtgatgggctgaacgtcaagaacggagtaaccatgaggtagctgtagatgtac                                                                                                            <  1:609460‑M1/301‑193 (MQ=255)`  
                                                                                                                                                                                                `gACACCAAGGACGTAATCCGCATCCTGCAAGCGCGTATCTATGAGCAATTAACGATTGACCTCTGGGAAGACGCAGAAGACTTGCTCAATGAATACTTGGAGGAAGTCGcacgacagaaagaaattgaccgcgctaaggcccgtaaagaacgtcacgaggggcgcttagaggcacgcagattcaaacgtcgcaaccgcaaggcacgtaaagcacacaaagctaagcgcgaaagaatgcttgctgcgtggcgatgggctgaacgtcaagaacggcgtaactatgaggtagctgtagatgtac                                                                                                            >  1:423484‑M1/1‑109 (MQ=255)`  
                                                                                                                                                                                                `gACACCAAGGACGTAATCCGCATCCTGCAAGCGCGTATCTATGAGCAATTAACGATTGACCTCTGGGAAGACGCAGAAGACTTGCTCAATGAATACTTGGAGGAAGTCGcacgacagaaagaaattgaccgcgctaaggcccgtaaagaacgtcacgaggggcgcttagaggcacgcagattcaaacgtcgcaaccgcaaggcacgtaaagcacacaaagctaagcgcgaaagaatgcttgctgcgtggcgatgggctgaacgtcaagaacggcgtaaccatgaggtagctgtagatgtac                                                                                                            <  1:615984‑M1/301‑193 (MQ=255)`  
                                                                                                                                                                                                `aacaccAAGGACGTAACCCGCATCCTGCAAGCGCGTAGCTATGGGCAATTAACGATTGACCTCTGGGAATACGCAGAAGACTTGCTCAATGAATACTTGGAGGAAGTCGcacgacagaaagaaattgaccgcgctaaggcccgtaaagaacgtcacgaggggcgcttagaggcacgcagattcaaacgtcgcaaccgcaaggcacgtaaagcacacaaagctaagcgcgaaagaatgcttgctgcgtggcgatgggctgaacgtcaagaacggcgtaaccatgaggtagctgtagatgtac                                                                                                            <  1:544066‑M1/300‑193 (MQ=255)`  
                                                                                                                                                                                                  `cacCAAGGACGTAATCCGCATCCTGCAAGCGCGTATCTATGAGCAATTAACGATTGACCTCTGGGAAGACGCAGAAGACTTGCTCAATGAATACTTGGAGGAAGTCGcacgacagaaagaaattgaccgcgctaaggcccgtaaagaacgtcacgaggggcgcttagaggcacgcagattcaaacgtcgcaaccgcaaggcacgtaaagcacacaaagctaagcgcgaaagaatgcttgctgcgtggcgatgggctgaacgtcaagaacggcgtaaccatgaggtagctgtagatgtacta                                                                                                          <  1:514315‑M1/301‑195 (MQ=255)`  
                                                                                                                                                                                                  `cacCAAGGACGTAATCCGCATCCTGCAAGCGCGTATCTATGAGCAATTAACGATTGACCTCTGGGAAGACGCAGAAGACTTGCTCAATGAATACTTGGAGGAAGTCGcacgacagaaagaaattgaccgcgctaaggcccgtaaagaacgtcacgaggggcgcttagaggcacgcagattcaaacgtcgcaaccgcaaggcacgtaaagcacacaaagctaagcgcgaaagaatgcttgctgcgtggcgatgggctgaacgtcaagaacggcgtaaccatgaggtagctgtagatgtacta                                                                                                          >  1:729685‑M1/1‑107 (MQ=255)`  
                                                                                                                                                                                                     `cAAGGACTTAATCCGCATCCGGCAAGCGCGTATCTATGAGCAATTAACGATTGACCTCTGGGAAGACGCAGAAGACTTGCTCAATGAATACTTGGAGGAAGTCGcacgacagaaagaaattgaccgagctaaggcccgtaaagaacgtcacgaggggcgcttagaggcacgcagattcaaacgtcgcaaccgcaaggcacgtaaagcacacaaagctaagcgcgaaagaatgcttgccgcgtggcgatgggctgaacgtcaagaacggcgtaaccatgaggtagctgtagatgtactagga                                                                                                       <  1:492611‑M1/301‑198 (MQ=255)`  
                                                                                                                                                                                                     `aagagacagaatccgcatccTGCAAGCGCGTATCTATGAGCAATTAACGATTGACCTCTGGGAAGACGCAGAAGACTTGCTCAATGAATACTTGGAGGAAGTCGcacgacagaaagaaattgaccgcgctaaggcccgtaaagaacgtcacgaggggcgcttagaggcacgcagattcaaacgtcgcaaccgcaaggcacgtaaagcacacaaagctaagcgcgaaagaatgcttgctgcgtggcgatgggctgaacgtcaagaacggcgtaaccatgaggtagctgtagatgtactagga                                                                                                       <  1:463911‑M1/292‑198 (MQ=255)`  
                                                                                                                                                                                                     `aagagacagaatccgcatccTGCAAGCGCGTATCTATGAGCAATTAACGATTGACCTCTGGGAAGACGCAGAAGACTTGCTCAATGAATACTTGGAGGAAGTCGcacgacagaaagaaattgaccgcgctaaggcccgtaaagaacgtcacgaggggcgcttagaggcacgcagattcaaacgtcgcaaccgcaaggaacgtaaagcacacaaagctaagcgcgaaagaatgcttgctgcgtggcgatgggctgaacgtcaagaacggcgtaaccatgaggtagctgtagatgtactagga                                                                                                       <  1:579824‑M1/292‑198 (MQ=255)`  
                                                                                                                                                                                                           `cgtaaaccaagtactgccagcgcgtatCTATGAGCAATTAACAATTGACCACTGGGAAGACGCAGAAGACTTGCTCAATGAATACTTGGAGGAAGTCGcacgacagaaagaaattgaccgcgctaaggcccgtaaagaacgtcacgaggggcgattagaggcacgcagattcaaacgtcgcaaccgtaaggcacgtaaagcacacaaagctaagcgcgaaagaatgcttgctgcgtggcgatgggctgaacgtcaagaacggcgtaaccatgaggtagctgtagatgtactaggaagagcc                                                                                                 <  1:507659‑M1/288‑204 (MQ=255)`  
                                                                                                                                                                                                               `aTCCGCATCCTGCAAGAGCGTATCTATGAGCAATTAACGATTGACCTCTGGGAAGACGCAGAAGACTTGCTCAATGAATACTTGGAGGAAGTCGcacgacagaaagaaattgaccgcgctaaggcccgtaaagaacgtcacgaggggcgcttagaggcacgcagattcaaacgtcgcaaccgcaaggcacgtaaagcacacaaagctaagcgcgaaagaatgcttgctgcgtggcgatgggctgaacgtcaagaacggcgtaaccatgaggtagctgtagatctgtctcttatacacatct                                                                                             >  1:565985‑M1/1‑94 (MQ=255)`  
                                                                                                                                                                                                                `ccctcatccTGCAAGTGCGTATCTTTGAGCAATTACCGATTGACCTCTGGGAAGACACAGAAGACTTGCTCAATGAATACTTGTAGGAAGTCGcacgacagaaagaaattgaccacgctaaggcccgtaaaggacgtcacgaggggcgcttagaggcacacagattcaaacgtcgcaaccgcaaggcgcgtaaagcacacaaagctaagcgcgaaagaatgcttgctgcgtggcgatgggctgaacgtcaagaacggcgtaaccatgaggtagctgtagatgtactaggaagaaccaataa                                                                                            <  1:603167‑M1/297‑209 (MQ=255)`  
                                                                                                                                                                                                                `accgCATCCTGCAAGCGCGTATCTATGAGCAATTAACGATTGACCTCTGGGAAGACGCAGAAGACTTGCTCAATGAATACTTGGAGGAAGTCGcacgacagaaagaaattgaccgcgctaaggcccgtaaagaacgtcacgaggggcgcttagaggcacgcagattcaaacgtcgcaaccgcaaggcacgtaaagcacacaaagctaagcgcgaaagaatgcttgctgcgtggcgatgggctgaacgtcaagaacggcgtaaccatgaggtagctgtagatgtactaggaagaaccaataa                                                                                            <  1:650035‑M1/300‑209 (MQ=255)`  
                                                                                                                                                                                                                   `ccaTCCTGCAAGCGCGTATCTATGAGCAAGTAACGATTGACCTCGGGGAAGACGAAGAAGAATTGCTCAATGAATACTGGGAGGAAGGAGaacgacagaaagaaattgaccgcgctaaggcccgtaaagaacgtcacgaggggcgcttagaggcacgcagattcaaacgtcgcaaccgcaaggcacgtaaagcacacaaagctaagcgcgaaagaatgcttgctgcgtggcgatgggctgaacgtcaagaacggcgtaaccatgaggtagctgtagatgtactaggaagaaccaataacgc                                                                                         <  1:265588‑M1/300‑212 (MQ=255)`  
                                                                                                                                                                                                                        `cTGCAAGCGCGTATCTATGAGCAATTAACGATTGACCTCTGGGAAGACGCAGAAGACTTGCTCAATGAATACTTGGAGGAAGTCGcacgacagaaagaaattgaccgcgctaaggcccgtaaagaacgtcacgaggggcgcttagaggcacgcagattcaaacgtcgcaaccgcaaggcacgtaaagcacacaaagctaagcgcgaaagaatgcttgctgcgtggcgatgggctgaacgtcaagaacggcgtaaccatgaggtagctgtagatgctgtctcttatacacatctccgagccc                                                                                    >  1:39469‑M1/1‑85 (MQ=255)`  
                                                                                                                                                                                                                          `gCAAGCGCGTATCTATGAGCAATTAACGATTGACCTCTGGGAAGACGCAGAAGACTTGCTCAATGAATACTTGGAGGAAGTCGcacgacagaaagaaattgaccgcgctaaggcccgtaaagaacgtcacgaggggcgcttagaggcacgcagattcaaacgtcgcaaccgcaaggcacgtaaagcacacaaagctaagcgcgaaagaatgcttgctgcgtggcgatgggctgaacgtcaagaacggcgtaaccatgaggtagctgtagatgtactaggaagaaccaataacgctatgctc                                                                                  <  1:136252‑M1/301‑219 (MQ=255)`  
                                                                                                                                                                                                                              `gcgcGTATCTATGAGCAGTTAACGATTGACCTCTGGGAAGACGCAGAAGACTTGCTCAATGAATACTTGGAGGAAGTCGcacgacagaaagaaattgaccgcgctaaggcccgtaaagaacgtcacgaggggcgcttagaggcacgcagattcaaacgtcgcaaccgcaaggcacgtaaagcacacaaagctaagcgcgaaagaatgcttgctgcgtggcgatgggctgaacgtcaagaacggcgtaaccatgaggtagctgtagatgtactaggaagaaccaataacgctatgctctggg                                                                              <  1:400756‑M1/301‑223 (MQ=255)`  
                                                                                                                                                                                                                               `ggcgtATCTATGAGCAATTAACGATTGACCTCTGGGAAGACGCAGAAGACTTGCTCAATGAATACTTGGAGGAAGTCGcacgacaaaaagaaattgaccgcgctaaggcccgtaaagaacgtcacgaggggcgcttagaggcacgcagattcaaacgtcgcaaccgcaaggcacgtaaagcacacaaagctaagcgcgaaagaatgcttgctgcgtggcgatgggctgaacgtcaagaacggcgtaaccatgaggtagctgtagatgtactaggaagaaccaataacgctatgctctgggt                                                                             <  1:472547‑M1/300‑224 (MQ=255)`  
                                                                                                                                                                                                                               `cgcgTATCTATGAGCAATTAACGATTGACCTCTGGGAAGACGCAGAAGACTTGCTCAATGAATACTTGGAGGAAGTCGcacgacagaaagaaattgaccgcgctaaggcccgtaaagaacgtcacgaggggcgcttagaggcacgcagattcaaacgtcgcaaccgcaaggcacgtaaagcacacaaagctaatcgcgaaagaatgcttgctgcgtggcgttgggctgaacgtcaagaacggcgtaatcatttgttagctgtagatgtactaggcagaatcaataactctatgctctgggt                                                                             >  1:559544‑M1/1‑78 (MQ=255)`  
                                                                                                                                                                                                                               `cgcgTATCTATGAGCAATTAACGATTGACCTCTGGGAAGACGCAGAAGACTTGCTCAATGAATACTTGGAGGAAGTCGcacgacagaaagaaattgaccgcgctaaggcccgtaaagaacgtcacgaggggcgcttagaggcacgcagattcaaacgtcgcaaccgcaaggcacgtaaagcacacaaagctaagcgcgaaagaatgcttgctgcgtggcgatgggctgaacgtcaagaacggcgtaaccatgaggtagctgtagatgtactaggaagaaccaataacgctatgctctgggt                                                                             <  1:236047‑M1/301‑224 (MQ=255)`  
                                                                                                                                                                                                                                    `ataagagacagattaacgattgaCCTCTGGGAAGACGCAGAAGACTTGCTCAATGAATACTTGGAGGAAGTCGcacgacagaaagaaattgaccgcgctaaggcccgtaaagaacgtcacgaggggcgcttagaggcacgcagattcaaacgtcgcaaccgcaaggcacgtaaagcacacaaagctaagcgcgaaagaatgcttgctgcgtggcgatgggctgaacgtcaagaacggcgtaaccatgaggtagctgtagatgtactaggaagaaccaataacgctatgctctgggtcaaca                                                                        <  1:309526‑M1/290‑229 (MQ=255)`  
                                                                                                                                                                                                                                     `tCTATGAGCAATTAACGATTGACCTCTGGGAAGACGCAGAAGACTTGCTCAATGAATACTTGGAGGAAGTCGcacgacagaaagaacttgaccgcgctaaggcccgtaaagaacgtcacgaggggcgcttagaggcacgcagattcaaacgtcgcaaccgcaaggcacgtaaagcacacacagctaagcgcgaaagcatgcttgctgcgtggcgctgggctgaacgtcaagaacggcgtaaccatgaggtcgctgtagctgtactaggaagaaccactaacgctatgctctctgtctctta                                                                       >  1:743951‑M1/1‑72 (MQ=255)`  
                                                                                                                                                                                                                                             `cAATTAACGATTGACCTCTGGGAAGACGCAGAAGACTTGCTAAATGAATACTTGGAGGAAGTCGcacgacagaaagaaattgaccgcgataaggcccgtaaagaacgtcacgaggggcgcttagaggcacgcagattcaaacgtcgcaaccgcaaggcacgtaaagcacacaaagctaagcgcgaaagaatgcttgctgcgtggcgatgggctgaacgtcaagaacggcataaccatgaggtagctgtagatgtactaggaagaaccaataacgctatgctctgggtcaacatgttctctg                                                               <  1:632457‑M1/301‑238 (MQ=255)`  
                                                                                                                                                                                                                                               `aTTAACGATTGACCTCTGGGAAGACGCAGAAGACTTGCTCAATGAATACTTGGAGGAAGTCGcacgacagaaagaaattgaccgcgctaaggcccgtaaagaacgtcacgaggggcgcttagaggcacgcagattcaaacgtcgcaaccgcaaggcacgtaaagcacacaaagctaagcgcgaaagaatgcttgctgcgtggcgatgggctgaacgtcaagaacggcgtaaccatgaggtagctgtagatgtactaggaagaaccaataacgctatgctctgggtcaacatgttctctggg                                                             >  1:200815‑M1/1‑62 (MQ=255)`  
                                                                                                                                                                                                                                                `ttAACGATTGACCTCTGGGAAGACGCAGAAGACTTGCTCAATGAATACTTGTAGGAAGTCGcacgacagaaagaaattgaccgcgctaaggcccgtaaagaacgtcacgaggggcgcttagaggcacgcagattcaaacgtcgcaaccgcaaggcacgtaaagcacacaaagctaagcgcgaaagaatgcttgctgcgtggcgataggctgaacgtcaagaacggcgtaaccatgaggtagctgtagatgtactaggaagaaccaataacgctatgctctgggtcaacatgttctctgggg                                                            <  1:486050‑M1/301‑241 (MQ=255)`  
                                                                                                                                                                                                                                                  `aaCGATTGACCTGTGGGAAGACGAAGAAGACTTGCTCAATTAATACTTGGAGGAAGTCGcacgacagaaagaaattgaccgcgctaaggcccgtaaagaacgtcacgaggggcgcttagaggcacgcagattcaaacgtcgcaaccgcaaggcacgtaaagcacacaaagctaagcgcgaaagaatgcttgctgcgtggcgatgggctgaacgtcaagaacggcgtaaccatgaggtagctgtagatgtactaggaagaaccaataacgctatgctctgggtcaacatgttctctggggac                                                          <  1:150992‑M1/301‑243 (MQ=255)`  
                                                                                                                                                                                                                                                         `gACCTCTGGGAAGACGCAGAAGACTTGCTCAATGAATACTTGGAGGAAGTCGcacgacagaaagaaattgaccgcgctaaggcccgtaaagaacgtcacgaggggcgcttagaggcacgcagattcaaacgtcgcaaccgcaaggcacgtaaagcacacaaagctaagcgcgaaagaatgcttgctgcgtggcgatgggctgaacgtcaagaacggcgtaaccatgaggtagctgtagatgtactaggaagaaccaataacgctatgctctgggtcaacatgttctctggggactttccgt                                                   >  1:408223‑M1/1‑52 (MQ=255)`  
                                                                                                                                                                                                                                                         `gACCTCTGGAAAGACGCAGAAGACTTGCTCAATGAATACTTGGAGGAAGTCGcacgaaagaaagaaattgaccgcgctaaggcccgtaaagaacgtcacgaggggcgcttagaggcacgcagattcaaacgtcgcaaccgcaaggcacgtaaagcacacaaagctaagcgcgaaagaatgcttgctgcgtggcgatgggctgaacgtcaagaacggcgtaaccatgaggtagctgtagatgtactaggaagaaccaataacgctatgctctgggtcaacatgttctctggggactttaagg                                                   <  1:552144‑M1/301‑250 (MQ=255)`  
                                                                                                                                                                                                                                                           `ccTCTGGGCAGACGCAGAAGACTTGCTCAATGAATACTTGGAGGAAGTCGcacgacagaaagaaattgaccgcgctaaggcccgtaaagaacgtcacgaggggcgcttagaggcacgcagattcaaacgtcgcaaccgcaaggcacgtaaagcacacaaagctaagcgcgaaagaattgttgctgcgtggcgatgggcttaacgtccaagacggcgtaaacatgagggagctgtagatgtacgaagaagaacaaataacgctatgctctgggtcaagatcttccctggggactttacgtcg                                                 >  1:41656‑M1/1‑50 (MQ=255)`  
                                                                                                                                                                                                                                                                              `gaCTTGCTCAATGAATACTTGGAGGAAGTCGcacgacagaaagaaattgaccgcgctaaggcccgtaaagaacgtcacgaggggcgcttagaggcacgcagattcaaacgtcgcaaccgcaaggcacgtaaagcacacaaagctaagcgcgaaagaatgcttgttgcgtggcgatgggctgaacgtcaagaacggcgtaaccatgaggtagctgtagatgtactaggaagaaccaataacgctatgctctgggtcaacatgttctctggggactttaaggcgcttgaggaacgaatcgcgc                              <  1:341263‑M1/301‑271 (MQ=255)`  
                                                                                                                                                                                                                                                                                   `gCTCAATGAATACTTGGAGGAAGTCGcacgacagaaagaaattgaccgcgctaaggcccgtaaagaacgtcacgaggggcgcttagaggcacgcagattcaaacgtcgcaaccgcaaggcacgtaaagcacacaaagctaagcgcgaaagaatgcttgctgcgtggcgatgggctgaacgtcaagaacggcgtaaccatgaggtagctgtagatgtactaggaagaaccaataacgctatgctctgggtcaacatgttctctggggactttaaggcgcttgaggaacgaatcgcgctgcac                         <  1:722742‑M1/301‑276 (MQ=255)`  
                                                                                                                                                                                                                                                                                        `ataagagacaggaggaagtcgcacgacagaaagaaattgaccgcgctaaggcccgtaaagaacgtcacgaggggcgcttagaggcacgcagattcaaacgtcgcaaccgcaaggcacgtaaagcacacaaagctaagcgcgaaagaatgcttgctgcgtggcgatgggctgaacgtcaagaacggcgtaaccatgaggtagctgtagatgtactaggaagaaccaataacgctatgctctgggtcaacatgttctctggggactttaaggcgcttgaggaacgaatcgcgctgcactggcg                    <  1:660099‑M1/291‑281 (MQ=255)`  
                                                                                                                                                                                                                                                                                           `aaTACTTGGAGGAAGTCGcacgacagaaagaaattgaccgcgctaaggcccgtaaagaacgtcacgaggggcgcttagaggcacgcagattcaaacgtcgcaaccgcaaggcacgtaaagcacacaaagctaagcgcgaaagaatgcttgctgcgtggcgatgggctgaacgtcaagaacggcgtaaccatgaggtagctgtagatgtactaggaagaaccaataacgctatgctctgggtcaacatgttctctggggactttaaggcgcttgaggaacgaatcgcgctgcactggcgtaa                 <  1:271413‑M1/301‑284 (MQ=255)`  
                                                                                                                                                                                                                                                                                            `aTACTTGGAGGAAGTCGcacgacagaaagaaattgaccgcgctaaggcccgtaaagaacgtcacgaggggcgcttagaggcacgcagattcaaacgtcgcaaccgcaaggcacgtaaagcacacaaagctaagcgcgaaagaatgcttgctgcgtggcgatgggctgaacgtcaagaacggcgtaaccatgaggtagctgtagaggtactaggaagaaccaataacgctatgctctgggtcaacatgttctctggggactttaaggcgcttgagggacgaatcgcggtgcactggcggtgt                >  1:302011‑M1/1‑17 (MQ=255)`  
                                                                                                                                                                                                                                                                                             `tACTTGGAGGAAGTCGcacgacagaaagaaattgacctcgctaaggcccgtaaagaacgtcacgaggggcgcttagaggcacgcagattcaaacgtcgcaaccgcaaagcacgtaaagcacacaaagctaagcgcgaaagaatgcttgctgcgtggcgatgggctgaacgtcaagaacggcgtaaccatgaggtagctgtagatgtactcggaagaaccaataacgctatgctctgggccaacatgttctctggggactttaaggcgcttgaggaacgcatcgcgctgcactggcgtcatg               >  1:229853‑M1/1‑16 (MQ=255)`  
                                                                                                                                                                                                                                                                                              `aCTTGGAGGAAGTCGcacgacagaaagaaattgaccgcgctaaggcccgtaaagaacgtcacgaggggcgcttagaggcacgcagattcaaacgtcgcaaccgcaaggcacgtaaagcacacaaagctaagcgcgaaagaatgcttgctgcgtggcgatgggctgaacgtcaagaacggcgtaaccatgaggtagctgtagatgtactaggaagaaccaataacgctatgctctgggtcaacatgttctctggggactttaaggcgcttgaggaacgaatcgcgctgcactggcgtaatgc              <  1:192539‑M1/301‑287 (MQ=255)`  
                                                                                                                                                                                                                                                                                              `aCTTGGAGGAAGTCGcacgaaagaaagaaattgaccgcgctaaggccagtaaagaacgtcacgaggggcgcttagaggcacgcagattcaaacgtcgcaaccgcaaggcacgtaaagcacacaaagctaagcgcgaaagaatgcttgctgcgtggcgatgggctgaacgtcaagaacggcgtaaccatgaggtagctgtagatgtactaggaagaaccaataacgctatgctctgggtcaacatgttctctggggactttaaggcgcttgaggaacgaatcgcgctgcactggcgtaatgc              <  1:286941‑M1/301‑287 (MQ=255)`  
                                                                                                                                                                                                                                                                                               `tttgGAGGAAGTCGcacgacagaaagaaattgaccgcgctaaggcccggaaagaacgtcacgaggggcgcttagaggcacgcagattcaaacgtcgcaaccgcaaggcacgtaaagcacacaaagctaagcgcgaaagaatgcttgctgcgtggcgattggctgaacgtcaagaacggcgtaaccatgaggtagctgtagatgtactaggaagaaccaataacgctatgctctgggtcaacatgttctctggggactttaaggcgcttgaggaacgaatcgcgctgcactggcgtaatgcc             <  1:358356‑M1/300‑288 (MQ=255)`  
                                                                                                                                                                                                                                                                                                  `ggaggaAGTCGcacgacagaaagaaattgaccgcgctaaggcccgtaaagaacgtcacgaggggcgcttagaggcacgcagattcaaacgtcgcaaccgccaggcacgtaaagcacacaaagctaagcgcgaaagaatgcttgctgcgtggcgatgggctgaacgtcaagaacggcgtaaccatgcggtatctgtagatgtactaggaagaaccaataacgctatgctctgggtcaacatgttctctgggtcctttacggcgcttgaggcactaatcgctcttcactgtcgtactgctgac          >  1:751045‑M1/1‑11 (MQ=255)`  
                                                                                                                                                                                                                                                                                                          `tCGcacgacagacagaaattgaccgcgctaaggcccgtaaagaacgtcacgaggggcgcttagaggcacgcagattcaaacgtcgcaaccgcaaggcacgtaaagcacacaaagctaagcgcgaaagaatgcttgctgcgtggcgatgggctgaacgtcaagaacggcgtaaccatgaggtagctgtagatgtactaggaagaaccaataacgctatgctctgggtcaacatgttctcttgggcctttaaggcgcttgcggaacgaatcgcgctgcacttgcgtaatgccgaccggatggc  >  1:785330‑M1/1‑3 (MQ=255)`  

                                                                                                                                                                                                                                                                                                            `|`                                                                                                                                                                                                                                                                                                            
`AACGTTTTCGACCACGCTTACGAAATGCTGAAAGAAAACATCCGTTATGATGACATCCGTGACACTGATGACCTGCACGATGCTATTCACATGGCTGCCGATAATGCAGTTCCGCACTACTACGCTGACATCTTTAGCGTAATGGCAAGTGAGGGCATTGACCTTGAGTTCGAAGACTCTGGTCTGATGCCTGACACCAAGGACGTAATCCGCATCCTGCAAGCGCGTATCTATGAGCAATTAACGATTGACCTCTGGGAAGACGCAGAAGACTTGCTCAATGAATACTTGGAGGAAGTCGAGGAGTACGAGGAGGATGAAGAGTAATGTCTACTACCAACGTGCAATACGGTCTGACCGCTCAAACTGTACTTTTCTATAGCGACATGGTGCGCTGTGGCTTTAACTGGTCACTCGCAATGGCACAGCTCAAAGAACTGTACGAAAACAACAAGGCAATAGCTTTAGAATCTGCTGAGTGATAGACTCAAGGTCGCTCCTAGCGAGTGGCCTTTATGATTATCACTTTACTTATGAGGGAGTAATGTATATGCTTACTATCGGTCTACTCACCGCTCTAGGTCTAGCTGTAGGTGCAT  >  NC_001604.1/952‑1550`

  

Alignment Legend

Aligned base mismatch/match (shaded by quality score): `ATCG/ATCG` `< 3 ≤` `ATCG/ATCG` `< 11 ≤` `ATCG/ATCG` `< 24 ≤` `ATCG/ATCG` `< 36 ≤` `ATCG/ATCG` `< 38 ≤` `ATCG/ATCG`

Unaligned base: `atcg`    Masked matching base: `atcg`    Alignment gap: `‑`    Deleted base: `‑`
